# A mechanistic model of infection: why duration and intensity of contacts should be included in models of disease spread

**DOI:** 10.1186/1742-4682-6-25

**Published:** 2009-11-17

**Authors:** Timo Smieszek

**Affiliations:** 1 Institute for Environmental Decisions, Natural and Social Science Interface, ETH Zurich, Universitaetsstrasse 22, 8092 Zurich, Switzerland

## Abstract

**Background:**

Mathematical models and simulations of disease spread often assume a constant per-contact transmission probability. This assumption ignores the heterogeneity in transmission probabilities, e.g. due to the varying intensity and duration of potentially contagious contacts. Ignoring such heterogeneities might lead to erroneous conclusions from simulation results. In this paper, we show how a mechanistic model of disease transmission differs from this commonly used assumption of a constant per-contact transmission probability.

**Methods:**

We present an exposure-based, mechanistic model of disease transmission that reflects heterogeneities in contact duration and intensity. Based on empirical contact data, we calculate the expected number of secondary cases induced by an infector (i) for the mechanistic model and (ii) under the classical assumption of a constant per-contact transmission probability. The results of both approaches are compared for different basic reproduction numbers *R*_0_.

**Results:**

The outcomes of the mechanistic model differ significantly from those of the assumption of a constant per-contact transmission probability. In particular, cases with many different contacts have much lower expected numbers of secondary cases when using the mechanistic model instead of the common assumption. This is due to the fact that the proportion of long, intensive contacts decreases in the contact dataset with an increasing total number of contacts.

**Conclusion:**

The importance of highly connected individuals, so-called super-spreaders, for disease spread seems to be overestimated when a constant per-contact transmission probability is assumed. This holds particularly for diseases with low basic reproduction numbers. Simulations of disease spread should weight contacts by duration and intensity.

## Background

Research has shown that the arrangement of potentially contagious contacts among the individuals of a society is a determining factor of disease spread: Both the repetition and the clustering of contacts diminish the size of an outbreak compared to a random mixing model [1-3]. Further, the epidemic threshold is low if the degree distribution shows a high dispersion [4,5]. In contrast to the vast body of literature that exists on the importance of network structure, only little emphasis has been put on the *quality *of such potentially contagious contacts, i.e. how long they last and how intensive they are. In fact, mathematical models and computer simulations of disease propagation often assume a constant per-contact transmission probability [cf. [4], [6], e.g.: [7-10]]. This approach ignores that, for instance, a short random encounter of two persons on a public bus is less likely to transmit a certain communicable disease than a rendezvous that lasted several hours.

Treating all contacts equally may lead to an overestimation of the individual transmission probability in cases of short, non-intense contacts and an underestimation in cases of intense, prolonged contacts. Allowing for heterogeneous transmission probabilities may then affect the model behaviour in various ways (e.g., altering the shape of the epidemic curves or changing the predictions of the effectiveness of intervention measures). In particular, the valuation of certain "risk groups," such as so-called super-spreaders defined as highly connected individuals [6], may change.

Several authors have already introduced heterogeneous transmission probabilities in their models. To do so, field data was typically analysed statistically to extract differences due to age, the susceptible individuals' immune responses, the levels of infectiousness of the infectors, and different contact situations [11-14]. For instance, in their model for Ebola epidemics, Legrand et al. differentiated the infection potential of hospital, funeral, and community settings [15], while Ferguson et al. distinguished household and non-household contacts in their model for an influenza pandemic [14]. The disadvantage of such *a posteriori *statistical models is that they become invalid when their underlying determinants (e.g., how individuals interact with other individuals) change.

Only few epidemic simulations model infection processes mechanistically (i.e., based on an *a priori *model instead of purely statistical analysis) to determine the transmission probability of differing contact situations: Alexandersen et al. [16] and Sørensen et al. [17], for example, show that basing large scale simulation models on quantities, such as intensity and duration of an exposure to infectious material, is possible and expedient. Existing mechanistic transmission models applied in simulations of disease propagation focus almost exclusively on aerosol transmission, but do not cover transmission by droplets and physical contact ("close contact"). Hence, simple mechanistic models of close contact contagion that can be used in simulations of disease spread are needed.

This paper is intended to highlight why mechanistic models of disease transmission are needed, to provide an example of how they can be built, and to show how they differ from the often-used transmission model that assumes a constant per-contact transmission probability. The proposed mechanistic approach for including the heterogeneity of transmission probabilities into disease spread simulations concentrates exclusively on diseases that are transmitted via close contact between an infector and a susceptible individual. We build on the fundamental knowledge that the risk of disease transmission is not only a function of the infectivity of the infectious agent and the quality of the immune response but also of the host's exposure to a specific infectious agent [18,19]. Particularly, we present evidence suggesting that the common assumption that highly connected individuals act as super-spreaders [6,20,21] might be misleading.

## Methods and Material

In this section, we first describe a formula that models transmission probabilities based on mechanistic considerations. Then, we introduce and describe an empirical data set of self-reported contacts qualified to transmit infectious disease. This data set was used to test the impact of the proposed transmission model. Finally, we introduce the scheme that describes how the outcome of both transmission models, i.e., the proposed mechanistic model assuming exposure dependency and the classical model assuming equally weighted contacts, were compared. Subsequently, we will refer to the first transmission model as the "mechanistic model" and the second model as the "classical model."

### A mechanistic transmission model

The probability of contracting a disease is closely linked to exposure to infectious organisms. A susceptible individual can only become infected if she/he is exposed to infectious organisms. Thereby, the transmission probability increases with an increase in the number of infectious organisms to which a susceptible individual is exposed. Subsequently, we refer to exposure as the cumulative, average amount of infectious medium ingested by a susceptible individual within a time period of interest due to close contact with an infectious person.

We base our proposed transmission model on the exponential relationship between the ingested dose and the infection risk as derived in Haas et al. [18] and used in several other publications [22-24]. Details describing how the following assumptions 1, 3, and 5 translate into an exponential dose-response model can be found in Haas et al. [18]. As an extension to this general formulation of an exponential relation between exposure and the risk of infection, we extrapolate the actual exposure from information about the duration and intensity of a contact between an infector and a susceptible individual. The proposed mechanistic model is based on the following underlying assumptions:

1. In principle, one infectious organism is sufficient to cause infectious disease. This hypothesis has been repeatedly supported by various studies against the alternative hypothesis assuming a threshold dose of infectious organisms must be passed to cause infection [19,25,26].

2. Every ingested infectious organism has a certain probability to survive until it reaches its target tissue and can initiate infection [18,27].

3. We assume that this survival probability is a constant, i.e., factors like the susceptible hosts' immune responses are assumed to be equally effective for all individuals. This assumption is a simplification of reality since susceptibility is known to differ between individual hosts [28]. However, for the purpose of this paper, such a simplification that keeps the model and the interpretation of its results manageable is justified.

4. The average dose of infectious material that is ingested by an individual is a linear function of the duration and intensity of the contact with an infectious individual. Research has shown that these measures are good predictors for individual attack rates of SARS [29]. In theory, we recognize that contact can be any kind of interaction between two individuals that is sufficient to exchange body fluids that can carry infectious particles. However, for reasons of manageability and measurability, we concentrate on conversational and physical contacts.

5. The actual amount of infectious organisms ingested by an individual follows a Poisson probability distribution with the average dose (defined in assumption 4) as parameter [18,19]. Thereby, we model the total (i.e., cumulative) average dose ingested during an entire simulation time step. This can lead to biased results in extreme cases [30], but given the fact that this assumption has proven to work well in the past and considering other uncertainties, utilizing this simplification is justified.

Based on this, the probability  that individual *n *becomes infected during simulation time step *t*_*x *_can be derived as(1)

where *I *is the total number of infectors;  [*s*^-1^] is the shedding rate (~microbial load) of infector *m *at simulation time step *t*_*x*_;  [1] is the contact intensity between the infector and the susceptible individual, which corresponds with the proportion of infectious material spread by infector *m *that is actually ingested by *n*; and  [*s*] is the time individuals *n *and *m *actually interact during time step *t*_*x*_. Finally, Θ is a calibration parameter that accounts for all relevant factors that are not explicitly represented, such as survival probability of the infectious agent. Simulation models can be fitted to measured epidemiological data, such as epidemic curves, or to targeted reproduction rates by means of Θ. We used Θ to achieve predefined reproduction rates for the contact structure introduced in the following section.

### Empirical contact structure

In the subsequently described test setting, empirical contact data is needed to compare the mechanistic transmission model with the classical one. We rely on contact data reported in a contact diary study that was conducted in Switzerland. A convenience sample of 54 participants was asked to report their potentially contagious contacts (as defined below) for 14 different days. Although a convenience sample is not representative for the whole population, the sample used here represents a very diverse cross-section of the population as can be seen in Table 1.

**Table 1:** Basic information about the sample and the contact structure

**Gender of participants**	
Female	27 (50.9%)
Male	26 (49.1%)
	
**Age distribution of participants**	
Mean and standard deviation	37.48 (*SD *= 16.71)
Min	20
25% percentile	24
50% percentile	29
75% percentile	52.25
Max	76
	
**Occupational status of participants^1^**	
Student	21 (39.6%)
Employed	35 (66.0%)
Neither student nor employed	11 (20.8%)
	
**Distribution of contact partners per day**	
Mean and standard deviation	9.92 (*SD *= 7.64)
Min	0
25% percentile	4
50% percentile	8
75% percentile	13
Max	51

^1 ^This does not sum up to 100% because multiple answers were possible

The design of the diary is similar to that used by Mikolajczyk et al. [31]. A potentially contagious contact is defined as (1) a mutual conversation of more than 10 words within a short distance (<2m), (2) physical contact in general, or (3) contact involving kisses. The participants were asked to categorize their contacts according to these three categories and to estimate how long they interacted with each reported contact person during an entire day based on six provided categories. However, for the analysis, we need concrete values instead of categories to calculate transmission probabilities as defined in Equation 1. Therefore, we assume a concrete duration, the arithmetic mean of the upper and lower bounds, for each category as given in Table 2.

**Table 2:** Time categories and translation into concrete values

Category in diary	Time value used for calculations
less than 5 min	2.5 min
5-15 min	10.0 min
15-60 min	37.5 min
1-2 h	90.0 min
2-4 h	180.0 min
more than 4 h	360.0 min

One diary had to be revoked due to deficient data quality; three of the remaining 53 participants provided only information for 5, 7, or 8 days, resulting in a total of 720 different person days with 7145 reported contact partners. In 36 of 7145 records, the information about contact duration was missing. These missing values were imputed based on probability distributions observed for the complete records. The processed data is provided in Additional File 1.

### Test setting for transmission models

In the results section, we compare how the proposed mechanistic transmission model differs from the classical model assuming an equal transmission probability for all contacts. Thereby, both the contact structure and the basic reproduction number *R*_0 _are fixed for both transmission models. We use the classical definition of *R*_0 _as the average number of secondary cases generated by an infected individual being introduced into a fully susceptible population [4].

We first analyse the effect of the observed patterns of contact duration and assume the intensities  to be equal and constant for all contacts. Then, we analyse the impact of contact intensity in a qualitative way. Information on shedding rates and inter-individual differences is available for many diseases (e.g., influenza cf. [14]). However, as we are more interested in exposure differences due to contact structure than in the impact of shedding rate differences, we also assume  to be equal for all infectors *m*. We further concentrate on hypothetical diseases with an infectious period of one day and basic reproduction numbers *R*_0 _= 1.5, 3.0, 4.5, and 6.0. With these assumptions, the contact intensity and the shedding rate can be included in a new calibration parameter , and Equation 1 can be simplified to(2)

The expected number of secondary cases *SC *generated by a specific infector *m *if introduced into a completely susceptible population can then be calculated as follows:(3)

with *S *represents the total number of susceptible individuals infector *m *has contact with during the day *m *is infectious. Finally, the equation(4)

reveals the basic reproduction number as defined previously when *X *includes the total population of interest.

The following two analyses are used to contrast the effect of the mechanistic model (Equation 2) against the classical model:

1) We illustrate the relationship between the expected number of secondary cases *SC *and the number of contacts *S *by calculating *SC *for the 720 person days as separate units of observation. *SC *is calculated according to Equations 2 and 3 and based on the contact durations measured with the contact diaries. We group the *SC*-values by *S *and show the so grouped *SC*-values in box plots. We do this for different values of Θ'; Θ' is determined such that *R*_0 _= 1.5, 3.0, 4.5, and 6.0 for the given test population according to Equation 4. The contact intensity *i *and the shedding rate *q *are assumed to be constants. We then compare the number of secondary cases *SC *of the simplified mechanistic model with the analogue *SC *value when the assumption of a constant per-contact transmission probability is used (also grouped by *S*).

2) In the second analysis, we calculate how the contact intensity is related to the duration of a contact in the empirical data set of potentially contagious contacts. This allows a qualitative discussion related to how the inclusion of variable contact intensities instead of a constant might affect the results found in analysis 1.

## Results

**Figure 1:**
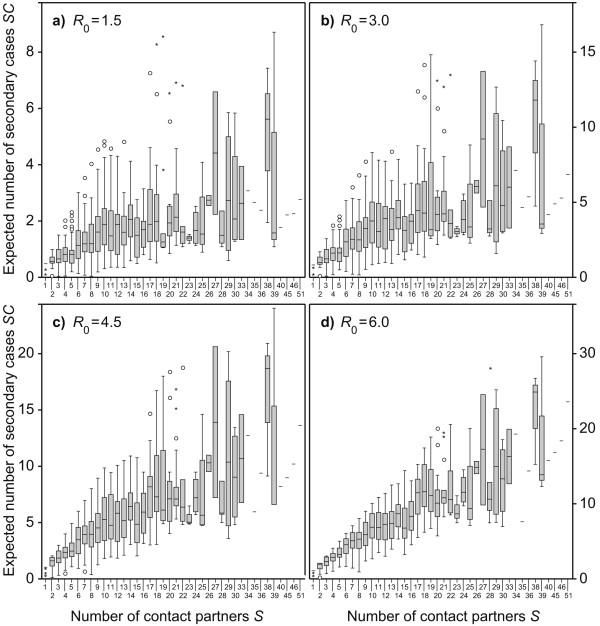
**Expected number of secondary cases versus number of contacts**. The boxplots show the distribution of the expected number of secondary cases that are induced by one infector that is introduced into a fully susceptible population. The values are grouped by the number of contact partners of the infectors. The boxes represent the interquartile range (*IQR*) with the median values marked as horizontal line. The whiskers are defined as max. ± 1.5·*IQR*. Circles are outliers and asterisks are extreme outliers. The subfigures represent the following basic reproduction numbers: a) *R*_0 _= 1.5; b) *R*_0 _= 3.0; c) *R*_0 _= 4.5; d) *R*_0 _= 6.0. Subfigures a-c are cropped such that one outlier lies outside the displayed range. The corresponding person day had 28 reported contacts and amounts *SC *= 14.15 for subfigure a, *SC *= 24.00 for b, and *SC *= 27.76 for c. The rationale for this outlier is presumably a reporting bias from the participant; i.e., the participant stated that she or he had close contact lasting for hours with a large number of persons at a festivity. Interacting closely with a large number of persons at a festivity over long time is almost impossible when the rigid contact definition in the diary is used.

Figures 1a-d show how the expected number of secondary cases of an infector introduced into a fully susceptible neighbourhood is related to the number of contacts (following Equation 3). Each subfigure represents another level of infectivity of the hypothetical infectious agent. Despite all of the random fluctuations, the following trends are quite clear:

1) Unsurprisingly, the expected number of secondary cases *SC *tends to be higher for highly connected individuals than for those with only few contacts.

2) For low contact numbers, the median expected number of secondary cases  and the number of susceptible contact partners *S* appear to be linearly related. For high contact numbers, the gradient /*S *is less steep than for low contact numbers.

3) As a disease becomes more infectious, the relationship between  and *S *seems to come closer to linearity. In Figure 1a (*R*_0 _= 1.5),  seems to reach a more or less stable plateau for *S *> 10, while in Figure 1d (R_0 _= 6.0),  appears to be an almost linear function of *S*. This impression is supported by regression analysis: If *S *is used as independent variable in a linear regression model to explain *SC*, the variance explained by this linear model equals 0.249 for *R*_0 _= 1.5, 0.339 for *R*_0 _= 3.0, 0.493 for *R*_0 _= 4.5 and 0.696 for *R*_0 _= 6.0 (all four linear regression analyses refuse the null hypothesis *R*^2 ^= 0 on a significance level of *p *< 0.01 using a F-test).

**Figure 2:**
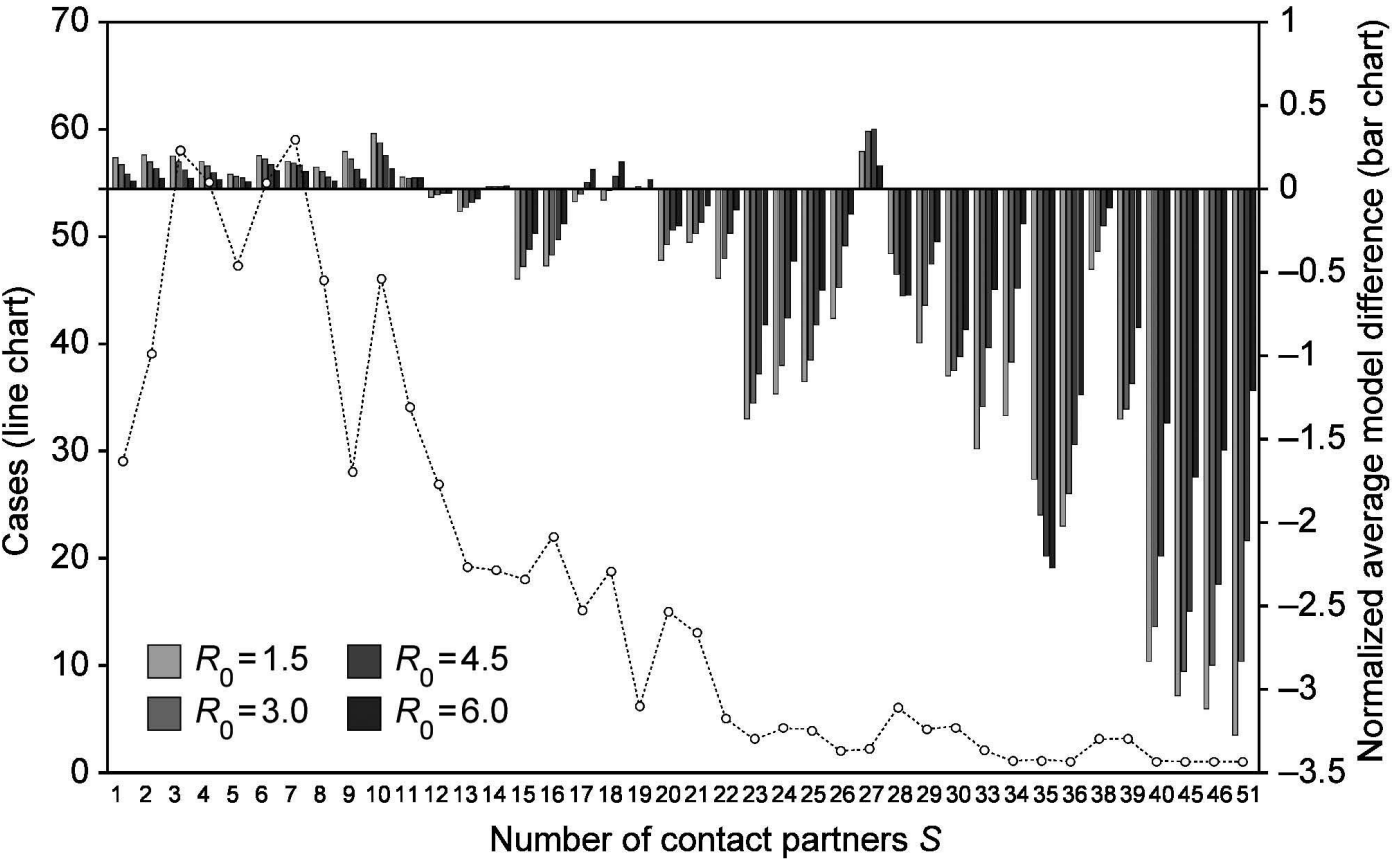
**Mechanistic versus classical model**. The bars show the average difference between the expected number of secondary cases of the mechanistic model *SC*_*mech *_and that of the classical model *SC*_*clas *_when normalized by the basic reproduction number *R*_0 _and grouped by the number of contact partners *S*. The sequence of reproduction numbers *R*_0 _within each category *S *goes from *R*_0 _= 1.5 on the left (light grey bars) to *R*_0 _= 6.0 on the right (black bars) in steps of 1.5. The line shows how many person days with exactly *S *contact partners exist in the sample.

Figure 2 shows how the proposed mechanistic model deviates from the classical transmission model if both are fitted to the same basic reproduction number and have the same underlying contact structure. Average deviations are shown for the whole range of *S *and *R*_0_. The average deviations were normalized by the basic reproduction number *R*_0_. Figure 2 reveals that individuals with less than 11 contacts have a slightly higher number of expected secondary cases when the transmission model depends on contact duration as compared to the case of a constant per-contact transmission probability. At the same time the classical model exceeds the mechanistic one in reference to highly connected individuals. The slight differences in case of individuals with less than 11 contacts can compensate for the rather pronounced differences of highly connected individuals as the majority of the person days reported eight or less contacts while highly connected individuals are rather seldom.

**Figure 3:**
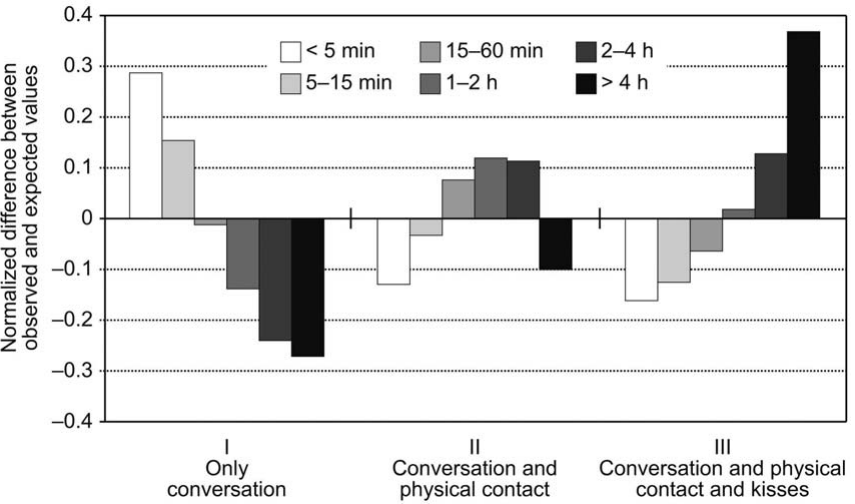
**Relationship between contact duration and intensity**. This figure shows the relationship between contact intensity (I, II, III) and duration (six categories). The bar charts result from subtracting the expected values (assuming no relation between intensity and duration of contacts) from the numbers of observations for each possible combination of duration and intensity. Hence, a positive value means that there were more observations of a certain duration-intensity-combination than would be expected if duration and intensity are independent. A negative value means that there were less observations than expected. The value zero means that the expected and observed numbers are the same. Every bar is normalized by the total number of observations for every time category.

Figures 1a-d and Figure 2 are based on Equation 2, which accounts for contact duration but ignores the influence of contact intensity. Figure 3 reveals how contact duration and contact intensity are interrelated, thereby allowing an interpretation of how the consideration of the contact intensity might alter the findings presented in Figures 1a-d and 2. Figure 3 shows separately for the three different levels of contact intensity how the reported numbers for the six duration categories deviate from the expected numbers (i.e., assuming no relation): Far more contacts of less then 5 minutes were observed than expected within the purely conversational contacts, while contacts of more than 4 hours are overrepresented in the most intensive contact category. This finding is also reflected in a positive correlation coefficient between these two ordinal variables: The non-parametric Kendall rank correlation results in *τ *= 0.388, which is significantly different from zero at the 0.01 level.

## Discussion

### Implications of the results presented

The presented results elucidate the implications of accounting for contact duration and intensity in simulations of disease spread. Figure 2 suggests that the importance of highly connected individuals, so-called super-spreaders, is strongly overestimated when all contacts are assumed to be equally likely to transmit infectious disease. This finding is particularly important because some well cited publications have concluded that highly connected individuals are major drivers of disease spread without accounting for the heterogeneity of the inter-individual transmission probabilities [20,21]. The results suggest that in the case of a disease with a low reproduction number, the expected secondary cases induced by individuals with many contacts are in the same range as those induced by individuals with medium numbers of contacts. Only when *R*_0 _is close to the mean number of contacts  is the expected number of secondary cases approximately linearly related to the number of contacts (see Figure 1 and the linear regressions shown in the results section).

This finding can be easily explained by the fact that the marginal total contact time decreases with every further contact person. In other words, most people have only a small set of persons (usually at home or at work) with whom they meet and interact for long periods during a day. Those individuals who meet with far more people than the average spend on average less time with every single contact person than those persons who have some or only a few contact partners per day. This can be illustrated with the example of train conductors, flight attendants, or supermarket cashiers; indeed, all of them have contact with hundreds of people a day, but they interact with each single contact only for a very short time.

As a consequence, highly connected individuals have more potentially contagious contacts than others, but these contacts are simultaneously on average less likely to transmit disease. Highly connected persons can reach their full "super-spreading" potential only if a disease is so contagious that almost every contact with a susceptible person leads to infection. Similar findings have been reported for sexually transmitted diseases: Research has shown that individuals with many different sexual partners per year are less important for disease propagation than often assumed because they have less sex acts per partner and in total than individuals with a smaller number of partners [32,33].

The conclusion that highly connected individuals are overestimated in their importance if a constant per-contact transmission probability is assumed is further supported by the analysis of the contact intensity as reported in the contact study. Theoretically, a short interaction between a susceptible and an infectious person could lead to a comparable amount of ingested infectious material as that of a long interaction assuming that the short interaction is more intensive than the long one. However, prolonged contacts tend to be more intensive than short contacts because they more often involve closer interaction, such as physical contact and kisses. This finding is plausible because those persons with whom the individuals spend much time are in most cases their loved ones, thereby indicating the higher likelihood of more intimate interactions than with casual acquaintances. As a result, the conclusions from the analysis of the pure contact durations are even further pronounced by taking contact intensities into account.

Therefore, our results suggest that sole concentration on the connectedness of individuals to explain super-spreading events is not valid. The explanation for super-spreading events might lie in a combination of many contacts and high shedding rates (cf. the notion of "super-shedder" [6]). Extreme numbers of secondary cases can only be achieved when the shedding rate  of an infector *m *is much higher than the average shedding rate and only if this infector *m *has many susceptible contacts *S*.

### Limitations

We see three limitations in our study. First, the empirical data set used to test the proposed exposure-based transmission model is rather small and not representative of the population. Furthermore, the person days used to calculate secondary cases are not statistically independent from each other as every person participating in the study contributed diary entries for several days. Finally, contact patterns are dependent on the cultural background and may look differently in Italy, Germany, Thailand, or Sudan [34]. Thus, the generalizability of our results may be questioned. Although this limitation exists, it is not likely to bias the presented results in a relevant manner. The observed contact patterns are plausible and theoretically grounded. An increasing number of contact partners per time unit naturally results in a decrease in the time spent with each single contact partner. Additionally, most people plausibly have only a very limited set of persons with whom they interact very closely. Additionally, the attributes of our contact structure are in complete agreement with other empirical studies on potentially contagious contacts that have also addressed similar attributes [34].

Secondly, the six time categories of the diary study offer rather imprecise information on the actual time that two persons interacted, and the three intensity categories are too vague to be translatable into concrete  values for use in Equation 1. Hence, the results presented have to be qualified in a quantitative rather than qualitative sense. For every time category, we defined a precise value (the arithmetic mean of the upper and lower boundaries) that was used for all calculations. However, a sensitivity analysis that alters the actual duration defined for every category within the given boundaries does not lead to qualitatively different results (see Additional File 2). Although the measured intensity indicator is not sufficiently precise to allow inclusion in a mathematical sense, the analysis clearly indicated that inclusion of contact intensity would amplify the observed phenomena rather than falsify our conclusions.

Finally, this paper makes statements about the expected number of secondary cases of infected individuals in a fully susceptible population. In a simulation model of disease spread, the importance of an individual also depends on her/his position within the contact network structure; i.e., the network position of every individual determines the likelihood of becoming infected as well as the susceptibility status of the surrounding individuals. Due to the complex nature of simulation models of disease spread, complete simulation models must be designed and tested for sensitivity to the changed transmission model proposed in order to allow precise statements on the impact of exposure-based transmission models on simulation outcomes.

## Conclusion

The goal of this paper was to provide evidence for the need of exposure-dependent transmission models and to suggest a mechanistic transmission model that can be used in simulations of disease spread. One remarkable result is that individuals with many contact partners seem to be less important for the transmission of diseases that are transmitted by droplets or physical contact than suggested by the classical assumption that all contacts are equally infectious. Particularly with only slightly infectious diseases, contacts should be differentiated by their potential to transmit infection when simulating disease spread.

This paper proposes an approach that enables the replacement of the problematic assumption of equally weighted contacts or purely statistical approaches to differentiate potentially contagious contacts with a mechanistic model. The proposed transmission model is based on well-established dose-response models that were developed in microbiology and builds upon assumptions that are closer to reality and better justifiable than the assumption that all contacts have the same transmission potential.

The spread of infectious disease is governed by a complex interplay of social and biological factors and to fully grasp its dynamics, processes on both the individual and the population level have to be understood [35,36]. Therefore we suggest including *a priory*, mechanistic models in simulations of disease spread and combining them with an *a posteriori*, statistical approach: Often data is available that allows fitting a simulation model that includes such mechanistic elements to empirical data, thereby making use of the advantages of both approaches.

## Competing interests

The author declares that he has no competing interests.

## Authors' contributions

TS is the sole author of this paper.

## Supplementary Material

Additional file 1 **Contact Data**. This additional file contains the processed empirical contact data that was used for calculating the results presented in this paper. Click here for file

Additional file 2 **Sensitivity Analysis**. We assumed the arithmetic mean of upper and lower bound as precise representation of the duration categories. In this additional file we provide an analysis on how sensitive the results react on changes to this assumption. Click here for file
