# Supplementary material for: A mechanistic model of infection: why duration and intensity of contacts should be included in models of disease spread
Source: Theor Biol Med Model. 2009 Nov 17;6:25. doi: 10.1186/1742-4682-6-25 (PMC2780993; doi:10.1186/1742-4682-6-25)
Supplement: Additional file 2 — Sensitivity Analysis. We assumed the arithmetic mean of upper and lower bound as precise representation of the duration categories. In this additional file we provide an analysis on how sensitive the results react on changes to this assumption. [file 1742-4682-6-25-S2.PDF]

Additional file – Sensitivity Analysis

The participants of the contact survey were asked to report the duration of their contacts in six distinct categories (cf. Table 2). For the analysis, we needed specific values rather than categories to calculate transmission probabilities (cf. Equation 2). We therefore assumed a concrete duration, the arithmetic mean of the upper and lower bounds, for each category as outlined in Table 2.

The aim of the following analysis is to test the sensitivity of the expected number of secondary cases to changes in the above-mentioned assumption. We calculate how the results change, if either the upper or the lower bound is chosen instead of the arithmetic mean. We choose the bounds as they are the extreme values of each category and thus cover the full range of possible assumptions. In case of the highest category (more than four hours), there is no upper bound. For that reason, we arbitrarily defined the extreme value for this category to be 480 minutes.

In the sensitivity analysis, we want to investigate how the average expected number of secondary cases (grouped by S) differs between the mechanistic and the classical model for varying duration assumptions. Consequently, we subtract the average value of SC_{clas} from the average value of SC_{mech} separately for each S. Each of the six duration categories can take one out of two possible values. As there are six categories, $2^6 = 64$ different combinations of duration assumptions have to be compared in the analysis. For the calculation of SC_{mech}, we define $\Theta' = 0.00195$ (cf. Equations 2 and 3 of the main text).

Figure S1 shows the results of the analysis described above. Each of the thin lines represents a unique combination of duration assumptions for the six duration categories. Data points above the zero line indicate that the mechanistic model in average leads to more secondary cases for the corresponding number of contact partners S than the classical model. Data points below the zero line indicate the opposite.

For low numbers of contact partners, there is a trend for all 64 combinations that the mechanistic model leads to higher numbers of secondary cases than the classical model. In contrast, the classical model exceeds the mechanistic model regarding the number of secondary cases for high numbers of contact partners. The most prominent deviations from this trend are found for $S = 27$. However, there are only two units of observation for $S = 27$, what makes this group of data points and other groups with many contact partners rather sensitive to outliers.

The results of all combinations are consistent with the results in the main text, which are based on the arithmetic mean of the upper and lower bounds.

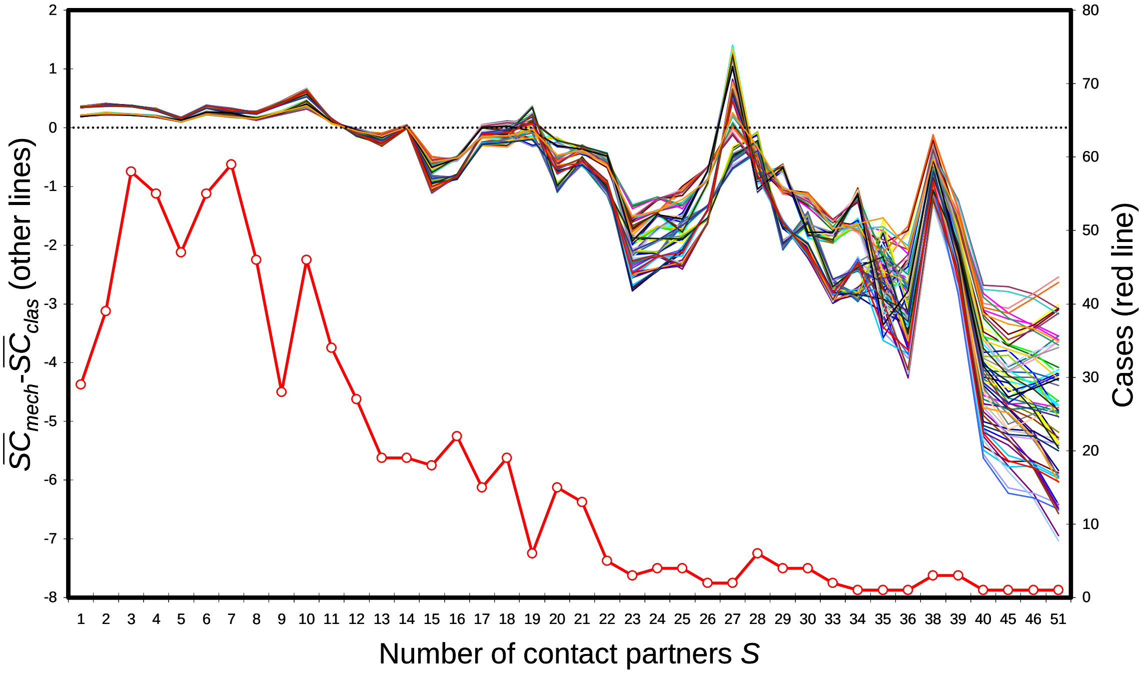

Figure S1 Results of the sensitivity analysis; thick, red line = number of cases in the sample with exactly S contact partners; other lines = $SC_{mech} - SC_{clas}$ **for all duration combinations.**
